# Supplementary material for: Dual-wavelength switchable single-mode lasing from a lanthanide-doped resonator
Source: Nat Commun. 2022 Apr 1;13:1727. doi: 10.1038/s41467-022-29435-w (PMC8975839; doi:10.1038/s41467-022-29435-w)
Supplement: Supplementary file 1 — Supporting Information [file 41467_2022_29435_MOESM1_ESM.pdf]

Supplementary Information
Dual-wavelength switchable single-mode lasing from a lanthanide-doped resonator

Limin Jin[#1†], Xian Chen[#2†], Yunkai Wu[#1], Xiangzhe Ai[2], Xiaoli Yang[1], Shumin Xiao[13†], Qinghai Song[13†]

[1]Ministry of Industry and Information Technology Key Lab of Micro-Nano Optoelectronic Information System, Harbin Institute of Technology, Shenzhen, 518055, P. R. China. [2]College of Materials Science of Engineering, Shenzhen University, Shenzhen 518060, P. R. China. [3]Collaborative Innovation Center of Extreme Optics, Shanxi University, Taiyuan, 030006, Shanxi, P. R. China.

#These authors contributed equally to this work: Limin Jin, Xian Chen, Yunkai Wu. Correspondence and requests for materials and data should be addressed to L.J. (email: jinlm2011@126.com), X.C. (email: x.chen87@outlook.com), S.X. (email: shumin.xiao@hit.edu.cn), or Q.S. (email: qinghai.song@hit.edu.cn)

Section-1. Synthesis and characterization of UCNCs

Reagents: $Y(CH_3CO_2)_3·xH_2O$ (99.9%), $Yb(CH_3CO_2)_3·xH_2O$, $Nd(CH_3CO_2)_3·xH_2O$ (99.9%), $Gd(CH_3CO_2)_3·xH_2O$ (99.9%), $Tm(CH_3CO_2)_3·xH_2O$ (99.9%), $Ho(CH_3CO_2)_3·xH_2O$ (99.9%), $Ho(CH_3CO_2)_3·xH_2O$ (99.9%), $Ca(CH_3CO_2)_2·H_2O$ (99.9%), NaOH (>98%), NH_4F (>98%), 1-octadecene (ODE) (90%), oleic acid (OA) (90%), were all purchased from Sigma-Aldrich. Absolute ethanol (99.85%), methyl alcohol (99.99%), and cyclohexane (99.9%) were purchased from J&K. All chemicals were used as received without further purification.

Synthesis of $NaGdF_4$:Yb/Nd core nanoparticles. The $NaGdF_4$:Yb/Nd core nanoparticle was synthesized by using our previously established methods. Firstly, 4-mL water solution of $RE(CH_3CO_2)_3$ (0.2 M, RE = Gd, Yb and Nd) was added to a binary solvent mixture of OA (8 mL) and ODE (12 mL) in a 50 mL flask. The mixture was heated at 160 °C for 60 min before cooling down to 45 °C. Thereafter, 6.5 ml of NH_4F (0.4 M) and 2 mL of NaOH (1 M) in methanol solution was added, and the resultant solution was stirred for 90 min. After the methanol was evaporated, the solution was heated to 300 °C under argon protection for 1 h and then cooled down to room temperature. The resulting nanoparticles in mixture were precipitated by addition of ethanol, collected by centrifugation at 5760 g for 3 min, washed with ethanol and methanol for 3 times, and finally re-dispersed in 4 mL cyclohexane for further use.

General procedure for the epitaxial growth of shell layers: The shell precursor was first prepared by mixing 4 mL water solution of corresponding lanthanide acetates (0.2 M) with 8 mL of oleic acid and 12 mL of 1-octadecene in a 50 mL flask followed by heating at 160 °C for 40 min. After cooling down to 50 °C, preformed core nanoparticles dispersed in 4 mL of cyclohexane were added along with a 5 mL methanol solution of NH_4F (1.5 mmol) and NaOH (1 mmol). The resulting mixture was stirred at 50 °C for 30 min, at which time the solution was heated to 290 °C under argon for 1 h and then cooled down to room temperature. The resulting nanoparticles were precipitated by addition of ethanol, collected by centrifugation at 5760 g for 5

min, washed with ethanol several times, and re-dispersed in 4 mL of cyclohexane. The procedures were repeated for layer-by-layer growth of multi-shell nanocrystals.

General sample characterizations: Powder X-ray diffraction (XRD) analysis was performed on a Bruker AXS D2 phaser with a graphite-monochromatized Cu Kα radiation (λ = 1.5406 Å). Low-resolution transmission electron microscopy (TEM) measurement was carried out on a FEI spirit T12 transmission electron microscope operating at an acceleration voltage of 120 kV. High-resolution TEM was performed on a FEI F30 transmission electron microscope operating at an acceleration voltage of 200 kV. Scanning electron microscopy (SEM) images were obtained from a Hitachi Model S-4700 scanning electron microscope with an accelerating voltage of 10-30 kV. The thickness of the film was analyzed by Dektak 150 thickness gauge. Photoluminescence spectra were recorded from cyclohexane dispersion of nanoparticles under the excitation of 808/980 nm diode lasers (20 W cm^{-2}).

Section-2. The proposed core-multishell UCNCs.

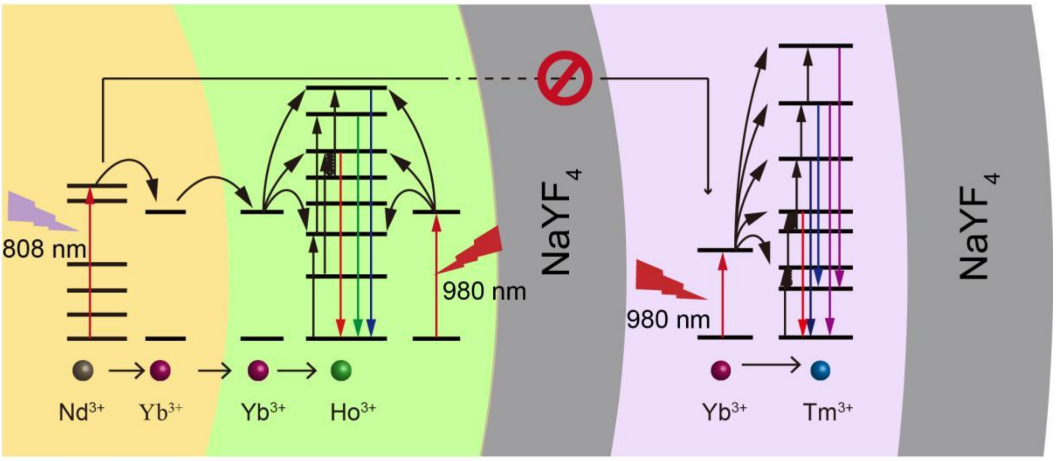

Figure S1 The proposed energy-mediated upconversion mechanism in the multishell nanocrystals under 808 nm (Nd^{3+}→Yb^{3+}→Ho^{3+}) and 980 nm (Yb^{3+}→Tm^{3+}) excitation, respectively. The distinct layers are highlighted by different colors.

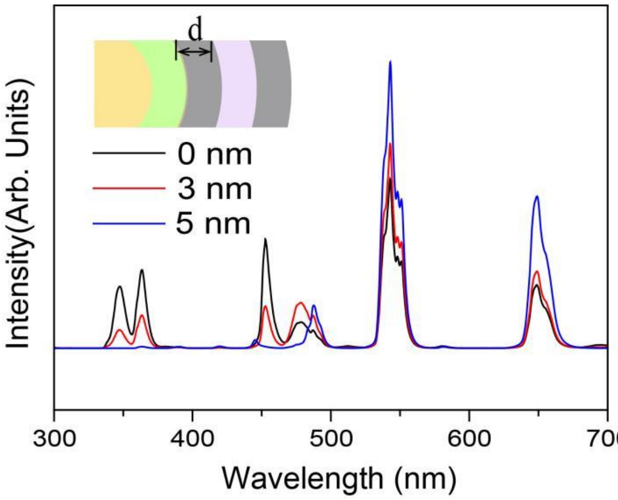

Figure S2 Emission spectra of the UCNCs with different shell thickness of $NaYF_4$:Ca intralayer under 808 nm excitation.

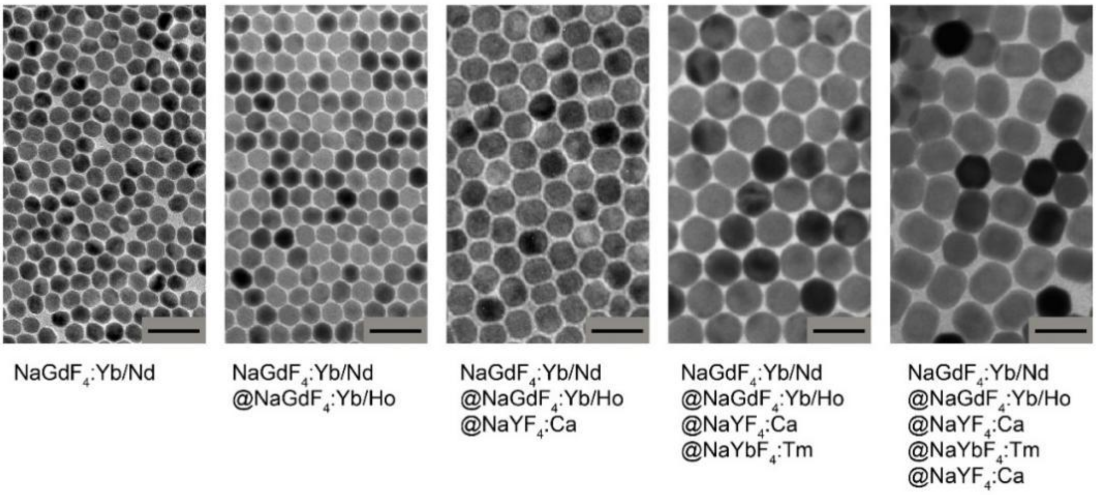

Figure S3 TEM images of the $NaGdF_4$:Yb/Nd(40/40)@$NaGdF_4$:Yb/Ho(48/2) @$NaYF_4$:Ca@$NaYbF_4$:Tm(1)@$NaYF_4$:Ca nanocrystals obtained at different stage of synthesis. The scale bars are 50 nm.

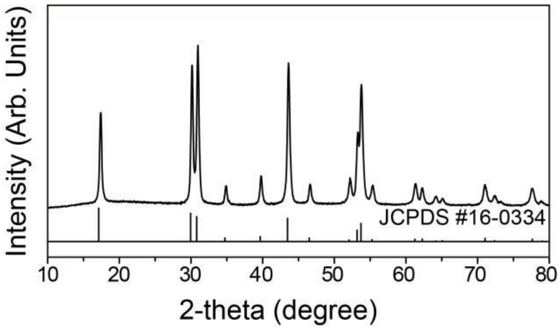

Figure S4 XRD pattern of the as-synthesized multi-shell UCNCs. The line spectrum is literature data for the hexagonal-phase $NaYF_4$.

Section-3. The UCNCs-based microdisk lasers.

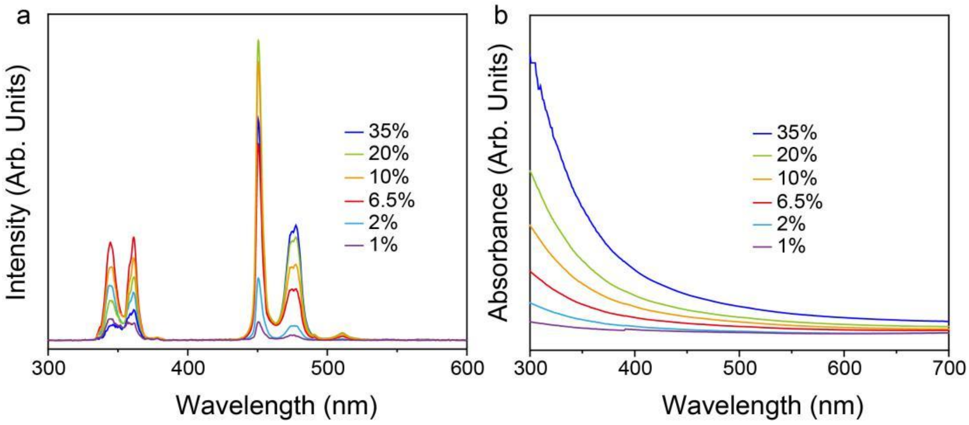

Figure S5 Optimizing the doping concentration of UCNCs in the PMs device. The dependence of (a) PL intensity under 980 nm pumping, and (b) the absorbance on the concentration of UCNCs in the cyclohexane solution.

As can be seen in Figure S5, the optimized doping concentration of UCNCs in the PMs device is determined by assessing the balance of the absorbance and the emission intensity of a set of solution containing varying concentrations of UCNCs. Although a low doping concentration of UCNCs can give rise to effective multiwavelength upconversion emission, a relative higher UCNCs concentration is needed to maximize the violet emission. However, the substantially higher UCNCs concentration (e.g. 10, 20, and 35%) significantly quenches the upconversion emission, especially at the wavelength of ~346 nm. Moreover, the increasing of UCNCs concentration deteriorates Q-factor of the whispering gallery cavity. Namely, it may play a negative role in the realization of single-mode lasing. Hence, the doping concentration of UCNCs was fixed at 6.5 wt% throughout the paper, unless otherwise specified.

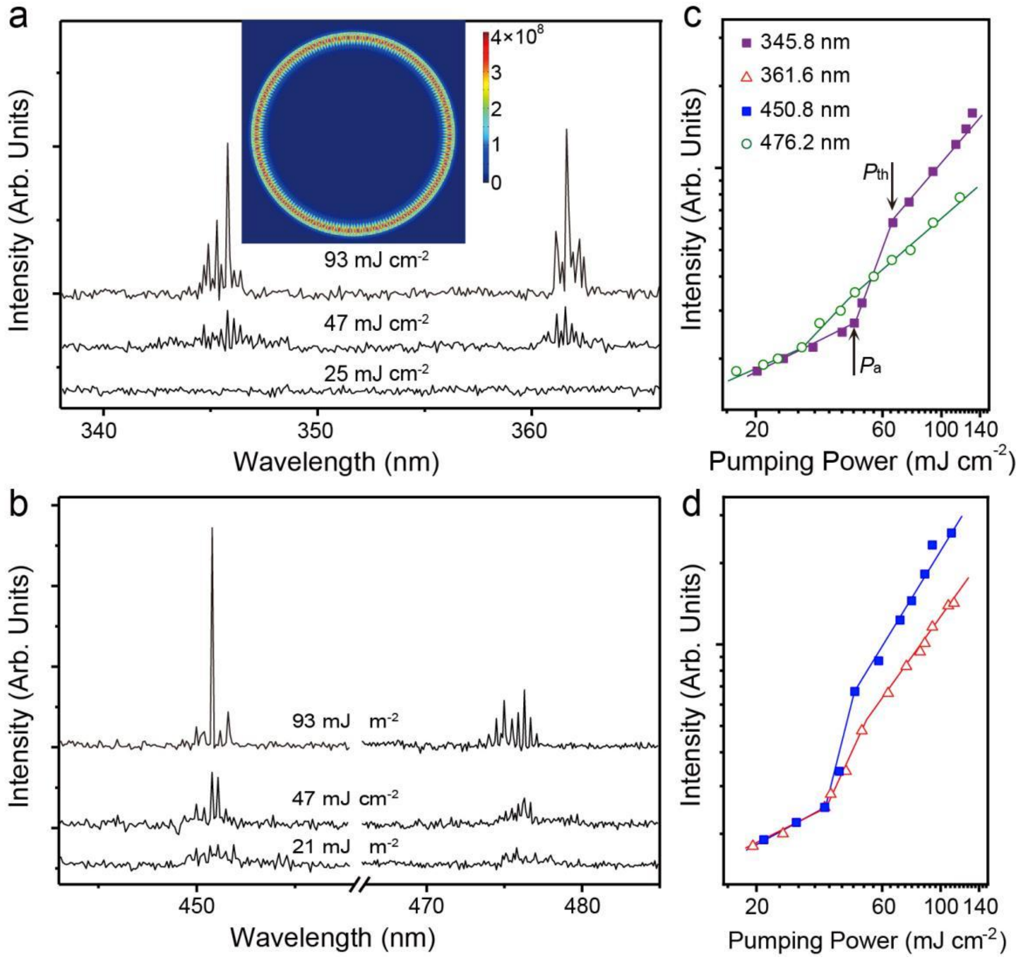

Figure S6 The lasing action of the UCNCs-based microdisk (d= ~100 μm, t= ~300 nm) under 980 nm excitation. (a,b) Emission spectra of the excited UCNCs-based microdisk at different power. The inset in S5(a) plots the numerical simulation of the excited microdisk (*i.e.*, d = 3 μm, n_{eff} = 1.58) in 2D geometry. (c,d) Lasing thresholds of four dominant peaks of the Tm^{3+} transitions. The solid lines are fitted for guidance.

Figure S6 gives the lasing characteristics of the excited UCNCs-based microdisk. The pumping light was directly focused onto the top surface of microdisk, with the emission light collected from the cavity boundary. The dependence of lasing spectra on the 980 nm power density were recorded in Figures S6(a, b). For low pumping power, four broad emission bands emerge from the spectra with the wavelengths centered at 345.8 nm, 361.6 nm, 450.8 nm, and 476.2 nm, respectively. As the pumping power increases, sharp peaks with periodic mode spacing (Δλ) ascend from six bands, with their intensity grow rapidly above the threshold values. The dependence of integrated output intensity on the pumping power (Figure S6(c)) exhibits an "S"-like shape with three regions of distinct slopes, implying the transition from spontaneous emission (i.e., < P_a) through amplification (i.e., $P_a < P < P_{th}$) to gain saturation (i.e., > P_{th}) (Figure S6(c-d)). The lasing threshold values are identified to be $P_{345.8nm}$ = 65.68, $P_{361.6\ nm}$ =50.62, $P_{450.8\ nm}$ = 49.75, and $P_{476.2\ nm}$ = 43.47 mJ cm^{-2}, respectively, as given by the second kink of the fitted lines (solid lines in Figures

S6(c,d)) from the corresponding light-light curves. It is observed that the experimental $\Delta\lambda$ values, recorded as $\Delta\lambda_{345.8\ nm} = 0.24$, $\Delta\lambda_{361.6\ nm} = 0.26$, $\Delta\lambda_{450.8\ nm} = 0.40$, and $\Delta\lambda_{476.2\ nm} = 0.44$ nm respectively, is in well accordance with the calculated values from the equation $\Delta\lambda = \lambda_0^2/n_{eff}L$. All these results unambiguously demonstrate multiwavelength lasing through the formation of whispering gallery modes (WGMs) along the boundary of the excited microdisk cavity, which is consistent with the simulation in the inset of Figure S6(a).

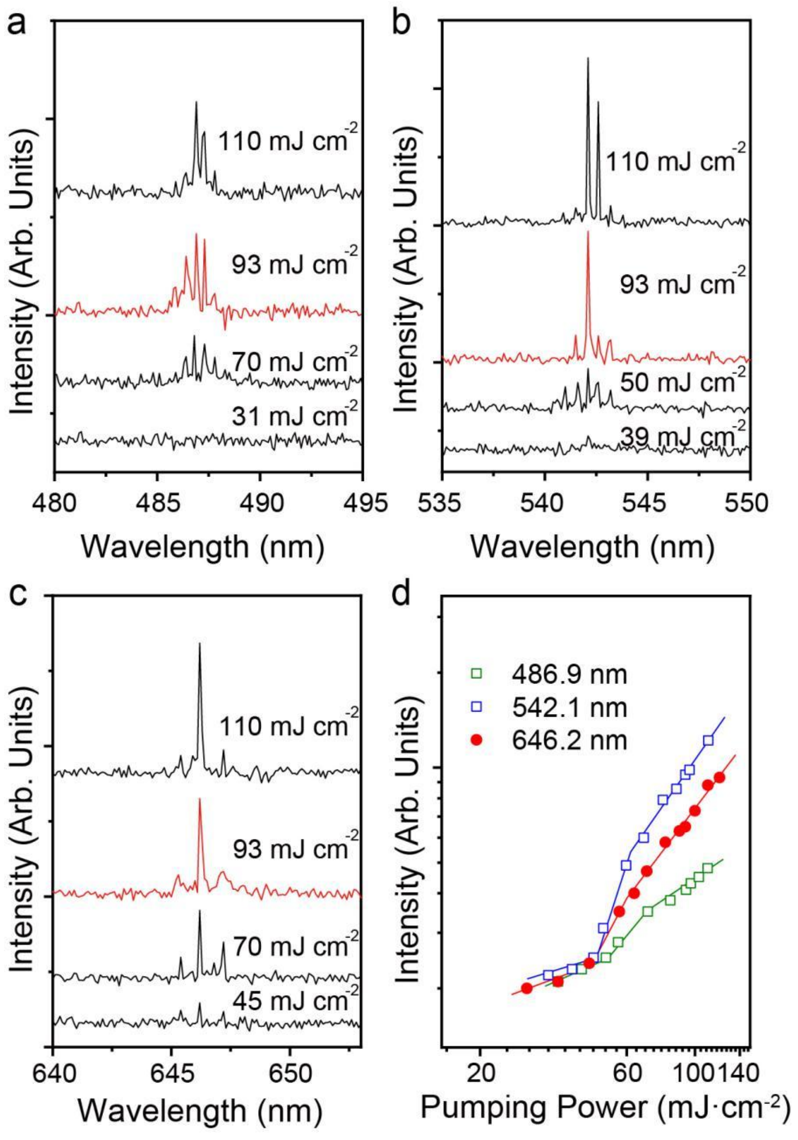

Figure S7 The lasing action of the UCNCs-based microdisk (d= ~100 μm, t= ~300 nm) under 808 nm pumping. (a-c) Emission spectra from the excited UCNCs-based microdisk at different power. (d) Lasing thresholds of three dominant peaks of the Ho^{3+} transitions. The solid lines are fitted for guidance.

Figures S7(a-c) plot the emission spectra in the excited UCNCs-based microdisk (d= ~100 μm, t= ~300 nm) as a function of 808 nm pumping power. The corresponding

light-light curves are shown in Figure S7(d). On increasing the pumping power, periodic sharp peaks centered at 486.9 nm, 542.1 nm and 646.2 nm occur from the broad emission bands and quickly dominate the spectra at high power density. In the experiment, the Δλ values read as $\Delta\lambda_{486.9\ nm}$ = 0.48 nm, $\Delta\lambda_{542.1\ nm}$ = 0.58 nm, and $\Delta\lambda_{646.2\ nm}$ = 0.85 nm, respectively, and the threshold values locate at $P_{486.9\ nm}$ = 70.98, $P_{542.1\ nm}$ = 61.94, and $P_{646.2\ nm}$ = 60.99 mJ cm^{-2}, respectively. Following the equation $\Delta\lambda = \lambda_0^2/n_{eff}L$ (*i.e.*, where n_{eff} = 1.58), the calculated L well matches the perimeter length of the UCNCs-doped microdisk with a diameter of 100 μm. Namely, the microdisk can simultaneously support the resonance of WGMs at blue, green and red bands, which verifies the multiwavelength lasing emission in the UCNCs-based microdisk.

Section-4. The UCNCs-based PMs lasers.

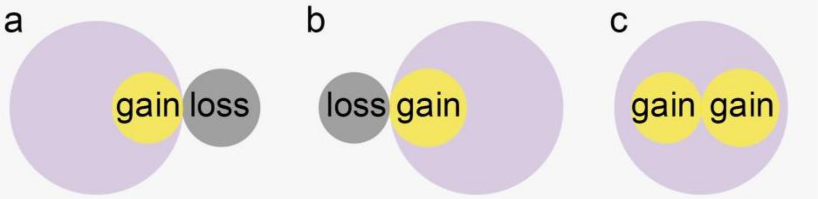

Figure S8 Three types of pumping configurations. (a) Left pumping, (b) right pumping, and (c) uniform pumping scheme. The gray, yellow and violet filled circles represent unexcited cavity, excited cavity and pumping light, respectively.

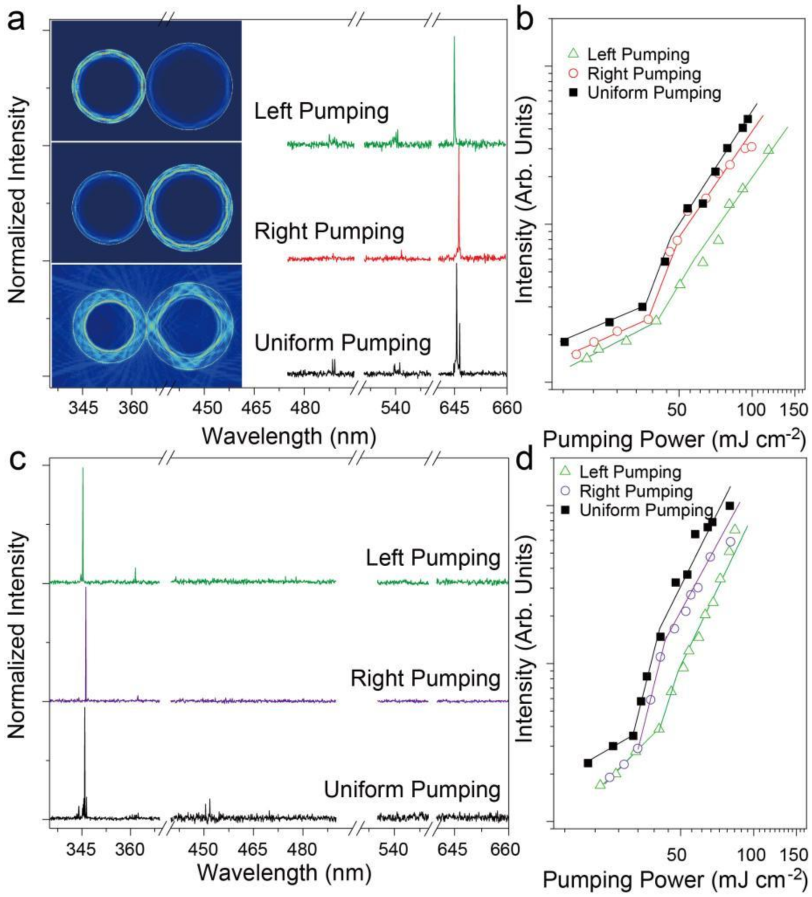

Figure S9 The PMs laser under three types of pumping configurations. Normalized emission spectra under (a) 808 nm, and (c) 980 nm left, right and uniform pumping with the corresponding light-light curves in (b,d) respectively. Solid lines are linear fitting to guide the eyes. From the L-L curves, the corresponding lasing threshold values reads as follows: (b) P_{left} = 47.52, P_{right} = 42.46, and $P_{uniform}$ = 41.01 mJ cm^{-2}, and (d) P_{left} = 57.03, P_{right} = 49.35, and $P_{uniform}$ = 46.25 mJ cm^{-2}, respectively. The insets give the corresponding numerical simulations of the excited PMs structure (d_{left} = 5.28 μm, d_{right} = 6.762 μm, and n_{eff} = 1.58 respectively).

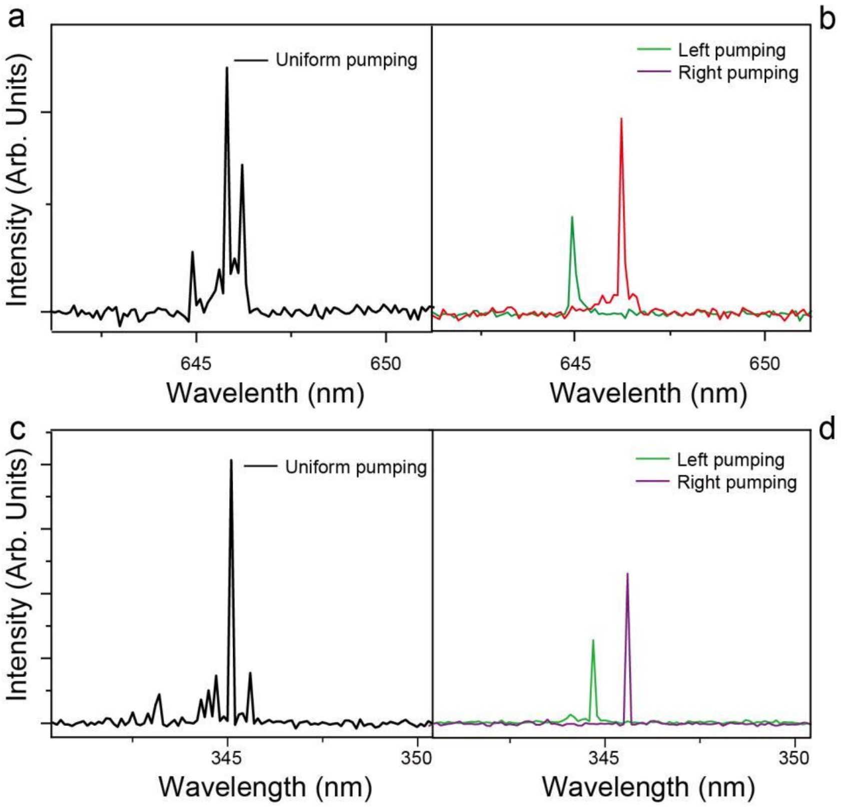

Figure S10 Lasing spectra of the excited PMs structure. Lasing spectra (a-b) under 808 nm, and (c-d) 980 nm right pumping at the power density of ~80 mJ cm^{-2}.

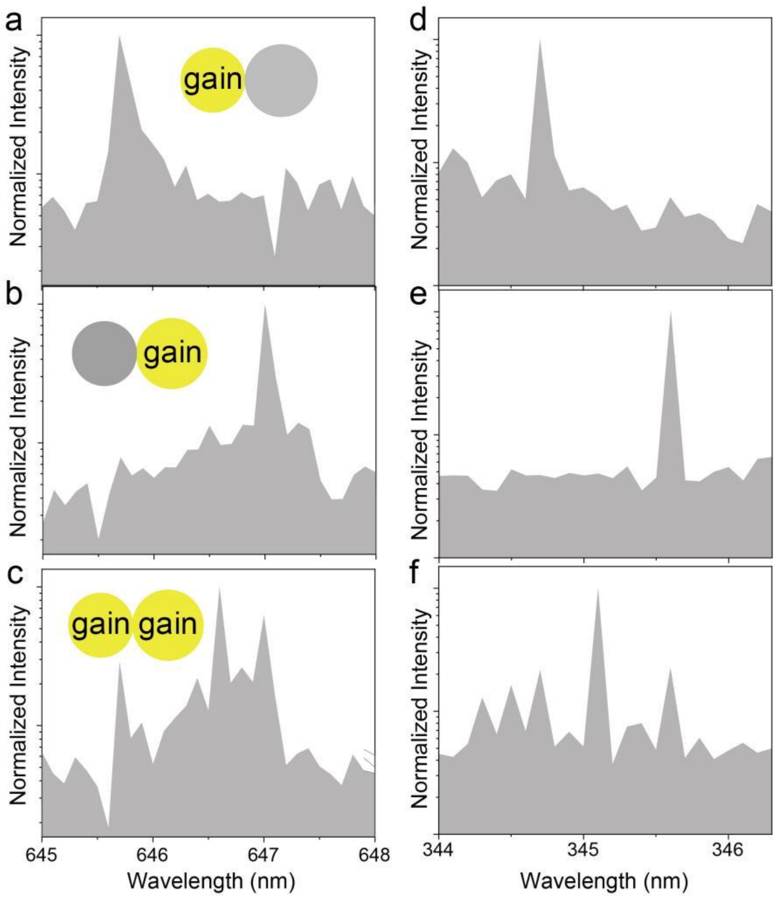

Figure S11 Experimental observation of single-mode lasing actions in the UCNCs-based PMs structure. (a-c) The corresponding lasing spectra in figure S5a with the wavelengths centered at 644.9, 646.2 and 645.6 nm under left, right and uniform 808 nm pumping, respectively. (d-f) The corresponding lasing spectra in figure S5c with the wavelengths centered at 344.7, 345.6 and 345.3 nm under left, right and uniform 980 nm pumping, respectively. It is observed that the emission spectra exhibits the single-mode action while only one of the constituent resonators is excited, whereas the emission spectra show conventional multimode lasing in uniformly pumped PMs structure. The gray and yellow filled circles represent unexcited cavity and excited cavity, respectively.

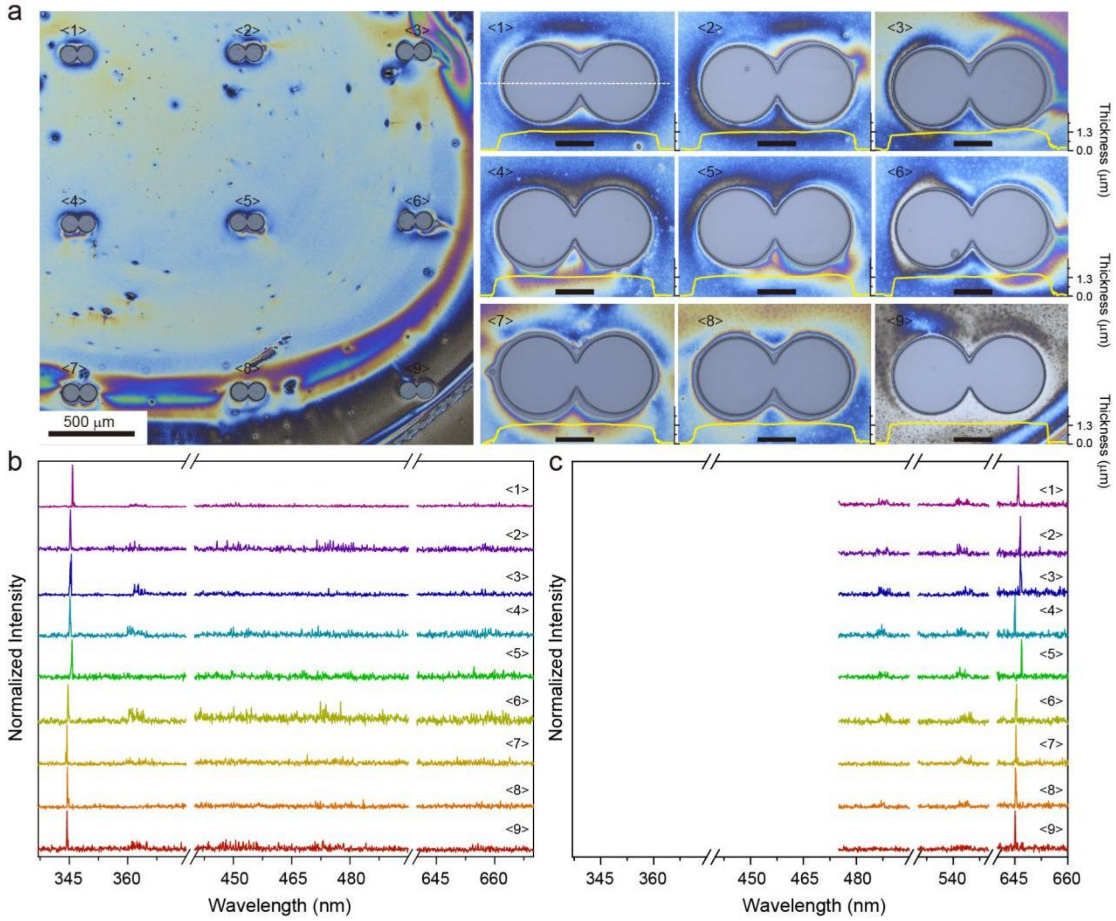

Figure S12 The short-listed PMs devices. (a) The optical images of another 3×3 UCNCs-based array (marked from <1> to <9>), and each PMs structure in the array. The surface roughness can be deduced from the thickness (the yellow profiles) observed along the central axis (see the white dotted line in <1>) of PMs device. The normalized lasing spectra of the short-listed PMs devices under the right excitation at (b) 980 nm, and (b) 808 nm, respectively. The pumping power is around 85 mJ cm^{-2}.

Another series of sample is characterized to confirm the device reproducibility. We can see the emission wavelengths of single-mode lasers are slightly different in nine neighboring PMs structures due to the fabrication inaccuracy. But their lasing spectral ranges well fall around 646 nm and 346 nm. These observations are consistent with the results in the main text even though the samples are fabricated in different times. Note that the large aggregates of UCNCs (marked by the orange arrows in <2> and <6>) at the boundary of PMs devices would significantly reduce the E-ratio of the resulting supermode. This is because the spoil of Q-factor would lead to the enhancement of spontaneous emission around the other peaks (i.e., ~362, ~451, ~476, ~487, and ~542 nm). But the dominant modes are still the ones at ~646 nm and ~346 nm. These results clearly show the good reproducibility of our devices.

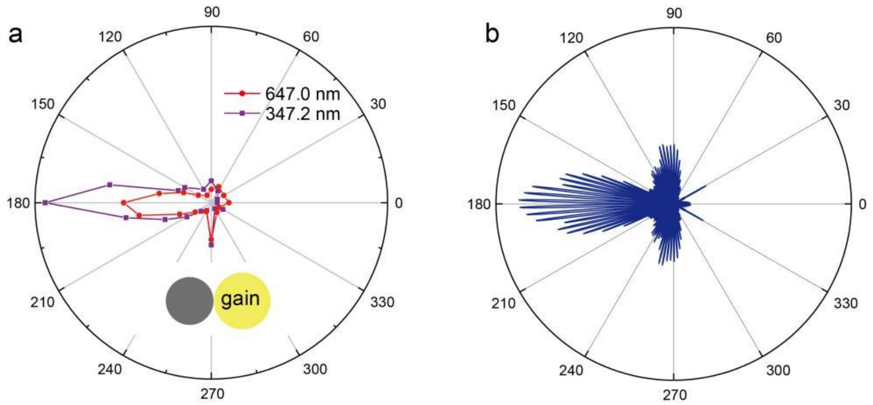

Figure S13 The unidirectional emission. (a) Far field patterns of the experimental lasing action at 646.2/345.6 nm under 808/980 nm right pumping, respectively. (b) The corresponding simulation with d_{left} = 5.28 μm, d_{right} = 6.762 μm, λ_0 = 646.2 nm, and n_{eff} = 1.58, respectively. The gray and yellow filled circles represent unexcited cavity and excited cavity, respectively.
